# Network for Development of Electroporation-Based Technologies and Treatments: COST TD1104

**DOI:** 10.1007/s00232-012-9493-8

**Published:** 2012-08-25

**Authors:** Damijan Miklavčič

**Affiliations:** Faculty of Electrical Engineering, University of Ljubljana, Tržaška 25, 1000 Ljubljana, Slovenia

**Keywords:** Electroporation, Cancer treatment, Pulsed electric field, Microbial inactivation, Food processing and preservation, Electrochemotherapy

## Abstract

Exposure of biological cells to a sufficiently strong external electric field results in increased permeability of cell membranes, referred to as “electroporation.” Since all types of cells (animal, plant and microorganism) can be effectively electroporated, electroporation is considered to be a universal method and a platform technology. Electroporation has become a widely used technology applicable to, e.g., cancer treatment, gene transfection, food and biomass processing and microbial inactivation. However, despite significant progress in electroporation-based applications, there is a lack of coordination and interdisciplinary exchange of knowledge between researchers from different scientific domains. Thus, critical mass for new major breakthroughs is missing. This is why we decided to establish cooperation between research groups working in different fields of electroporation. Cooperation in Science and Technology (COST), which funds networking and capacity-building activities, presents a perfect framework for such scientific cooperation. This COST action aims at (1) providing necessary steps toward EU cooperation of science and technology to foster basic understanding of electroporation; (2) improving communication between research groups, resulting in streamlining European research and development activities; and (3) enabling development of new and further development of existing electroporation-based applications by integrating multidisciplinary research teams, as well as providing comprehensive training for early-stage researchers. Results of this COST action will provide multiple societal, scientific and technological benefits from improving existing electroporation-based applications to adding new ones in the fields of medicine, biotechnology and environmental preservation.

## Introduction

Exposure of biological cells to a sufficiently strong external electric field results in transiently or permanently increased permeability of cell membranes, referred to as “electroporation.” Since all types of cells (animal, plant and microorganism) can be effectively electroporated, without addition of viral or chemical compounds, electroporation is considered to be a universal method and a platform technology. As such, it presents a common link to research areas in medicine, biotechnology, the environment and energy. The efforts needed to develop these applications and to address diverse aspects of electroporation (biological, chemical, physical, material science, modeling) are thus multidisciplinary. Therefore, to be efficient and successful, strong links need to be established between physicians, engineers and scientists working in different scientific domains. Electroporation is currently used in food and biomass processing (Martin-Belloso and Sobrino-Lopez 2011; Toepfl et al. 2007; Sack et al. 2010) and as a local treatment of cancer (Sersa et al. 2012; Testori et al. 2012). Preservation of food by electroporation was demonstrated to maintain color, flavor and levels of antioxidants, while destroying microorganisms. Recently, extraction of intracellular components from plants (Sack et al. 2010) as well as cryopreservation using electroporation (Phoon et al. 2008) have been demonstrated. Electroporation is also used for local cancer treatment of skin metastases, a treatment called “electrochemotherapy” (Marty et al. 2006). Since 2006, more than 4,000 patients in Europe have been successfully treated. The objective response rate of all tumors that were treated in these clinical trials was 78 %, with complete response of 52 % (Sersa et al. 2012). Clinical centers are already working on three innovative systems for drug delivery to the colorectal system, the brain, bone and liver metastases (Soden et al. 2007; Edhemovic et al. 2011; Mahmood and Gehl 2011; Agerholm-Larsen et al. 2011; Fini et al. 2010). However, for treatment of deep-seated tumor nodules, further technological developments are needed (Pavliha et al. 2012). Electroporation has shown potential in pretreatment of sludge and other substrates, leading to increased biogas production. Other applications, such as water treatment (Rieder et al. 2008; Gusbeth et al. 2009), extraction of oil from algae and of sugar from sugar beets (Loginova et al. 2011), gene therapy (Andre et al. 2008), DNA vaccination (Vandermeulen et al. 2009), cell fusion for systemic cancer treatment and human monoclonal antibody production (Usaj et al. 2010) are just a few of the foreseeable new applications.

Despite significant progress and increased use of electroporation-based applications, as well as the existence of a large number of nationally funded research projects, there is a lack of coordination and interdisciplinary exchange of knowledge between researchers from different scientific domains. Thus, critical mass in the form of human resources, knowledge from different domains and research facilities for major breakthroughs is often missing. Therefore, Cooperation in Science and Technology (COST) Action TD1104 has been prepared, which will provide the necessary steps toward cooperation in science and technology to foster basic understanding of electroporation and to enable further development of new and existing electroporation-based applications through integrating multidisciplinary research teams, as well as provide comprehensive training for students and early-stage researchers.

Classical EU-funded projects (e.g., EU FP, ERC, ESF, EUREKA) mainly support research and development, either basic or specific application-oriented. In contrast, COST funds networking and capacity-building activities, thereby representing a perfect framework for interdisciplinary international collaboration in electroporation research, which is at its current stage characterized by a relatively high level of national (54 %) and private (21 %) funding. Coordination of research in this action will at least partially address some of the global challenges (according to the Millennium Project, http://www.unmillenniumproject.org) associated with science and technology, health issues, long-term perspective, clean water and energy. Concrete societal, scientific and technological benefits from the action will arise in medicine (e.g., cancer treatment), biotechnology (e.g., extraction from algae and other cell cultures, improvement of industrial processes), environment preservation (e.g., wastewater treatment, biogas production, energy harvest from biomass) and food processing (e.g., extending shelf life of food, development of novel foods, obtaining new ingredients).

## About COST

COST was the first and is thus the oldest European network to coordinate nationally funded research activities. COST today covers 35 European member countries and is organized in nine scientific domains (for more details, see http://www.cost.esf.org/about_cost). The main goal of COST is to strengthen Europe in research through the support of cooperation and interaction between researchers. It has in all these years grown largely from being a European networking mechanism to being a wider, even worldwide, cooperation framework. COST provides funds for coordination of research through COST actions, such as this one on electroporation, while research remains funded on a national level. COST actions are thus networking activities based on predominantly nationally funded research projects on a research topic that is of interest to at least five COST countries. There is a great emphasis on gender balance and inclusion of young scientists. It also allows all interested scientists from countries who sign the Memorandum of Understanding (MoU, http://w3.cost.eu/fileadmin/domain_files/BMBS/Action_TD1104/mou/TD1104-e.pdf) to join the activities even after the start of the action.

The process of getting a specific COST action running involves several steps. Based on a call, which is announced twice per year, a researcher (the proposer was the author of this article) on behalf of other researchers interested in a topic submits a proposal for a COST action on a specific topic from one of eight domains. If the proposal spans multiple domains (like electroporation), then it is considered a transdomain proposal and is evaluated as such. In the call where the COST action “European network for development of electroporation-based technologies and treatments” was submitted more than 90 proposals were received. After the first evaluation, 11 proposers were asked to submit a full proposal. After the second evaluation, five proposers were invited to a hearing in front of the representatives of the domain committees, after which three proposals were selected for funding, for a success rate of little more than 3 %. The COST action on electroporation was submitted as a transdomain action spanning different domains from biomedicine and molecular biosciences; food and agriculture; information and communication technologies; material, physics and nanosciences; and earth systems science and environmental management (ESSEM) to chemistry and molecular sciences and technologies—thus a truly multidisciplinary, i.e., transdomain, COST action.

The networking activities within the COST action include scientific and administrative meetings, short-term scientific missions and organization of training schools. Meetings can be organized in the form of a management committee (i.e., administrative meeting), as a workshop on a specific topic or as a conference. Funding is available for the travel and subsistence costs of participants and for partial coverage of the organizing costs of the meeting. Short-term scientific missions are exchange visits of up to 3 months, aiming at strengthening collaboration between researchers from different countries. Emphasis is on supporting young researchers to learn new methods and perform experiments and measurements with equipment not available at their home institution. Training schools are intended for dissemination of research activities of the COST action and have to provide intensive training for young scientists in one of the laboratories but are also intended for retraining as a part of life-long learning. Funds are also available for dissemination of research results and publication through regular scientific publishing channels, such as peer-review journals, workshop and conference proceedings and books, but also general-information leaflets and brochures.

## Reasons for the Action

Despite significant progress and increased use of electroporation-based applications, there is a lack of coordination and interdisciplinary exchange of knowledge between researchers from different scientific domains. Furthermore, research and development of electroporation, even if funded within the EU program, is currently fragmented as it is very often only “a service” in developing new applications—critical mass is thus missing. In order to obtain the most from this platform technology and to avoid the same research being replicated and funded more than once, it is important to establish cooperation between research groups from different domains. This is why it is necessary to establish a coordination of research and network. The main reason for this action is to enable cross-fertilization of different areas that will allow exchange of experience and knowledge. This will facilitate collaboration of different specialists from engineering, physics, chemistry, biology and medicine, thus providing a multidisciplinary approach and critical mass for further improvement of existing and development of new electroporation-based applications in medicine, biotechnology, energy harvest from biomass and the food industry.

Immediate and future benefits of the action:

- New knowledge, closing the gap between basic research and applications
- Improving communication and avoiding duplication of research efforts
- New and improved medical treatments (cancer treatment, DNA vaccination, gene therapy)
- Safer and healthier food
- Savings in natural resources, development of energy-efficient processes
- Opening new markets and creating new jobs

Envisaged electroporation based applications developed within the action:

- New approaches in gene therapy for cancer treatment and DNA vaccination for the medical device industry
- In food processing, extraction of valuable ingredients, development of novel foods and improvement of industrial food processes in terms of food safety and food quality
- Microbial inactivation and biomass preprocessing in wastewater treatment, biogas production and energy production

## Planning and Preparing the Proposal

Experts working on electroporation and electroporation-based applications from different domains have been in contact, working occasionally on developing treatment or processing protocols but usually only on a national level and only exceptionally internationally within EU FP projects. Considerable efforts have been made in the past few years to bring together experts working with electroporation in different areas such as food processing, cancer treatment, pulsed power electronics and energy efficiency. However, the success was limited. Experts were meeting at conferences that were specialized, e.g., in cancer treatment in general, in food quality and processing or in bioelectrochemistry; but none of them gave the opportunity to focus on electroporation as a technology platform. Few experts in the field made an attempt to bridge the gap between different disciplines, but funding has been obtained predominantly on the national level, which potentially leads to funding duplication, poor coordination of research and difficulties in exploiting the results.

Establishing a new professional organization dedicated to the promotion of electroporation and electroporation-based applications through organization of dedicated conferences did not seem to be adequate at this stage of development and at this level of collaboration. Therefore, the COST action seems a perfect way to help the electroporation community to develop stronger links, exchange experience and bring together forces through establishing a communication platform including regular meetings.

During the COST action proposal an extensive questionnaire was prepared in order to collect relevant information regarding research groups and their funding. The questionnaire was divided into seven sections:

1. Introduction—Questionnaire for a new COST action
2. Personal and academic information
3. Scientific expertise
4. Facts and figures of laboratory/department
5. Participation in COST actions and other international cooperation
6. Expected results and benefits of the COST action
7. Additional information

**Fig. 1:**
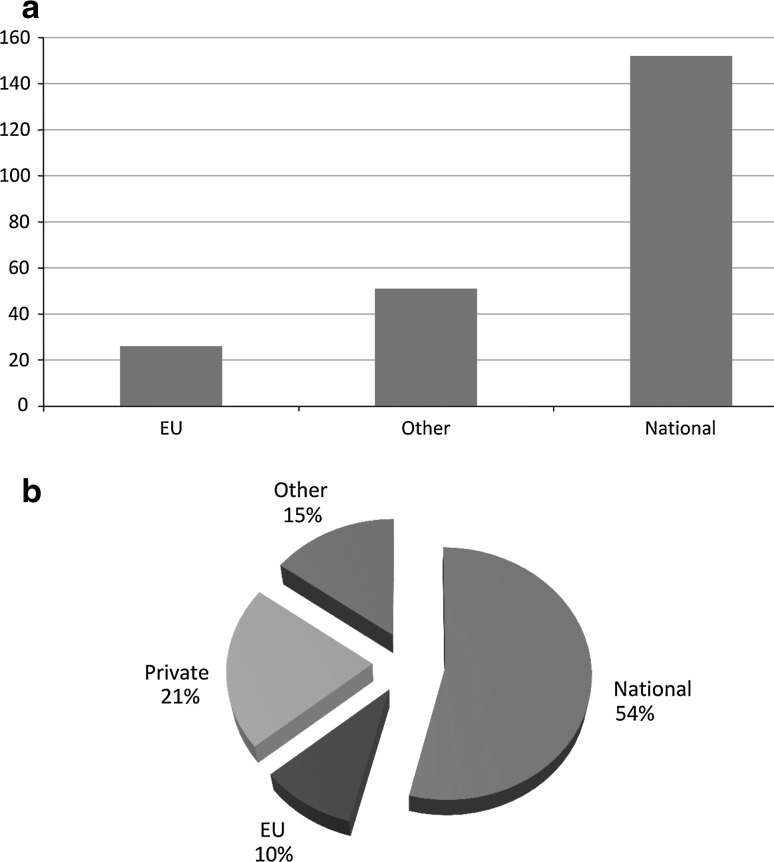
Number of projects on electroporation by type (**a**) and by sources of funding (**b**) conducted by project proposers in the last 3 years

This allowed us not only to prepare a high-quality proposal but also to collect data on existing projects on national, international and EU levels (Fig. 1a). The collected data will serve as a reference level for future research activities in the field of electroporation, against which the success of this COST action will be measured through the years of the action and at the end of the action.

A total of 73 experts from 25 countries participated in the preparation of this proposal. According to the data collected through the questionnaire, in the last 3 years these experts worked on a total of 229 projects related to electroporation, 152 of which were funded on a national level, with 26 EU projects and 51 other international projects. The funding of participating research groups comes predominantly from national (54 %), private (21 %) and EU (10 %) sources (Fig. 1b).

**Fig. 2:**
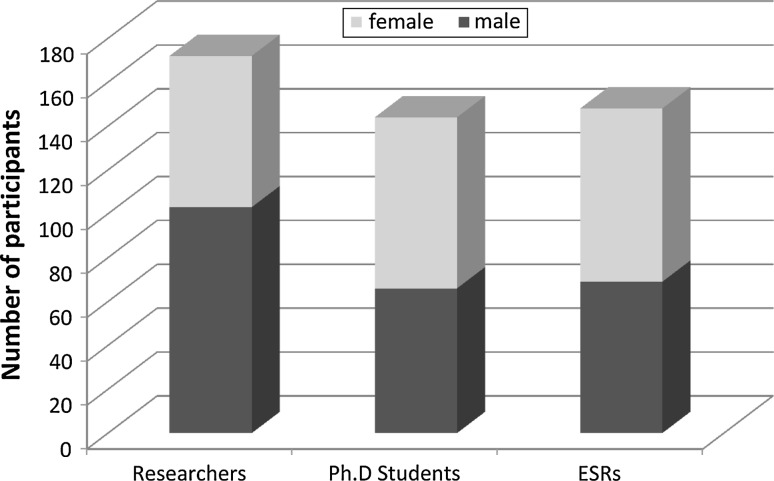
Number of individual researchers and their representation by sex and years in research

According to the responses to the questionnaire, it was established that 293 researchers and 115 PhD students are currently working in COST country laboratories. In addition to 56 research groups from 19 COST countries, 13 research groups with 56 researchers were from countries with reciprocal agreements (Argentina, Australia and New Zealand) and non-COST countries (Russian Federation, Ukraine and United States), and they also participated actively in the preparation of the action (see Fig. 2 for participant structure). In these research groups, 152 national and 51 international research projects in the field of electroporation were funded in the last 3 years. The groups also participated in 26 EU FP projects in the last 3 years. This alone provides the COST action with great potential for coordination of these projects, thus providing better use of resources, avoiding duplication of research and allowing synergistic effects to take place.

The number of countries (19 COST and 6 non-COST countries) that have actively participated in the preparation of the proposal clearly demonstrates the extent of existing research and the need for coordination of this research, which is predominantly funded on a national level (54 %). Starting the COST action will allow also “smaller” groups that are working “alone” or are just starting their research on a national level to join the network. Until the kick-off meeting that took place on April 10, 2012, in Brussels already 21 COST countries had signed the Memorandum of Understanding and had nominated national representatives.

## Work Plan of the Project

Improving existing and developing new applications of electroporation will be the topics of the research. Coordination of national and international projects will allow collaborations not only between teams working in specific fields of electroporation but also among partners from different disciplines by using multidisciplinary approaches. In particular, focus will be given to linking theoretical efforts (e.g., analytical and numerical modeling of electroporation) with experimental results to develop basic knowledge of this phenomenon, which will be incorporated into electroporation-based applications. Modeling efforts on different levels—from the molecular level (molecular dynamics simulations) to the cell and tissue level (finite element modeling)—will be linked vertically and validated with experimental results.

**Fig. 3:**
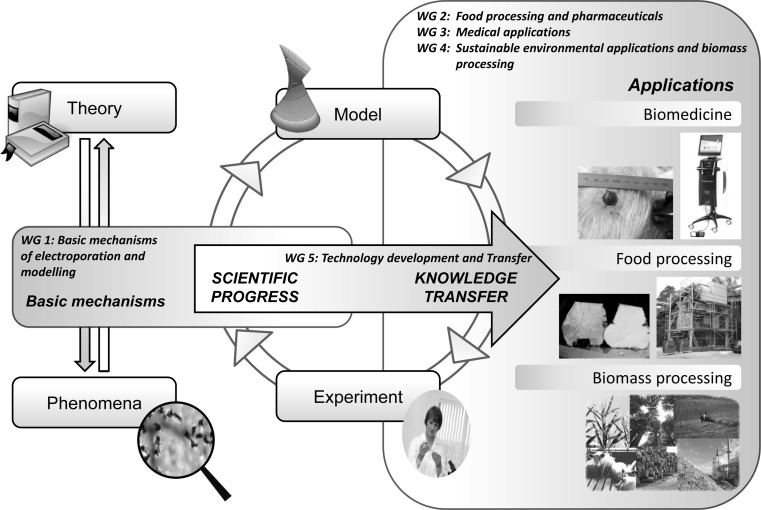
Diagram illustrating the research processes and knowledge transfer within the action, emphasizing the process of translating basic research into applications. The working groups (WGs) facilitate all stages of the process

The work plan of the action consists thus of investigating the basic mechanisms of electroporation and optimizing protocols in different electroporation-based applications, with special emphasis on food and biomass processing, medical applications and sustainable energy production (Fig. 3). Optimized protocols will allow technology development and transfer. The most important research tasks coordinated by the action are briefly described below.

1. Establishment of a close collaboration between researchers in biology, chemistry, physics, mathematics, informatics and engineering to enhance our understanding of electroporation and its associated processes. This will start immediately after approval of the action and will cover the first 3 years of the cooperative program. Emphasis will be on modeling the cell membrane with a goal of identifying the underlying processes of membrane electroporation and the associated phenomena (membrane destabilization, cytoskeleton reorganization, transmembrane pathways, etc.). Theoretical and modeling results will be compared to experimental data.
2. Optimizing electroporation protocols for industrial applications (e.g., food and biomass processing, microbial inactivation). Conditions used in these applications so far have only been determined empirically, while the latest developments, such as intracellular manipulation by nanosecond pulses, have not been implemented yet. Based on recent studies on a cellular level and basic research, processing conditions for industrial applications will be optimized with respect to low energy consumption. The knowledge about the impact of processing conditions on electroporation efficacy will be reviewed, taking into account specific industrial requirements (large-scale processing, high throughput, environmental and economic issues). General and application-oriented electroporation protocols for food and biomass processing will be gathered in a database. This will allow simple parameter selection for nonexperts and industrial end-users and provide an easy-to-access knowledge base for regulatory bodies.
3. Development of equipment design guidelines. Based on electroporation protocols, the guidelines for design of electroporation equipment (generators, electrode systems, microchambers) will be elaborated with the aim of achieving optimal treatment homogeneity, food safety and hygienic design. In addition, a database of applications already investigated and/or transferred into industry will be generated. The available scientific literature will be screened and information will be collected on equipment suppliers (e.g., product type, type of equipment, processing parameters). The database, accessible to COST partners via a Web site, will allow the selection of suitable, commercially viable application areas in food and biomass processing and the identification of new application fields.
4. Process monitoring. In the food industry, typically hazard analysis and critical control points concepts have to be followed to ensure food safety, where at critical points processing conditions are recorded. For electroporation, such protocols and suitable sensors still have to be developed. The identification of suitable monitoring techniques will be a key requirement for a broader application. Electrochemical reactions and electrode erosion should be minimized with regard to food safety. The impact of pulse wave shape and frequencies as well as other pulse characteristics and material-related effects will have to be determined to develop optimal equipment and select optimal processing conditions.
5. Development of electrochemotherapy (ECT) for cancer treatment. The development of electrodes for use in internal organs, identification and testing of new cancer drugs suitable for ECT and expansion of pulse parameters to increase versatility of ECT are challenges that still need to be addressed and that require future efforts. Clinical centers are already working on three innovative systems for drug and gene delivery to the colorectal system, the brain, bone and liver metastases. Several centers have worked with drugs other than conventional chemotherapeutic agents, and expansion of the spectrum of such drugs would be advantageous. The extension of attainable pulse parameter ranges should increase the flexibility of electroporation used in clinics in achieving both reversible electroporation (as used for drug delivery) and irreversible electroporation (with the aim of causing cell death by electroporation alone).
6. Expansion of delivery technology for large molecules, with a focus on end products such as DNA vaccines. Use of electroporation to transfer nucleic acids to various tissues (e.g., skeletal muscle, tumors, skin, liver) has benefited from the electrodes, generators and knowledge accumulated in the development of ECT protocols. In vivo, combinations of electroporating and electrophoretic pulses have been shown to be highly efficient, and clinical studies have demonstrated their safety compared to classical viral methods for gene therapy. The results of the clinical trials must now be promoted, and the researchers should dedicate their efforts to finding optimal protocols, developing adequate electrodes and generators, etc. The first clinical trials of gene electrotransfer (EGT) either have recently finished or are in progress. The range of applications of EGT is broad, and particularly promising are its applications in the treatment of cancer and infectious diseases. Development of nonviral EGT is a collective task, and the COST action will impact its emergence by gathering scientists with different expertise.
7. Identification of new effects of interaction of short electric pulses with biological matter and their possible applications. In particular, emphasis will be placed on the use of pulses with extremely short duration and high voltage. Recently, experimental evidence for growth stimulation of plants and fungi after treatment with pulses of nanosecond duration and sublethal amplitude was found, which promises applications in biomass production and conditioning. Within this task, further basic research demands will be identified.
8. Identification of synergetic process combinations. A combination of thermal and electroporation treatments has been shown to increase the microbial inactivation performance of electroporation-based treatment. In case of wastewater disinfection, the inactivation rate of the combined process was also higher than the sum of the rates of the single processes. Possibilities of synergetic process combinations for other applications will be investigated with regard to saving processing energy and simplifying existing processes.

## Organization and Implementation of the Project

**Fig. 4:**
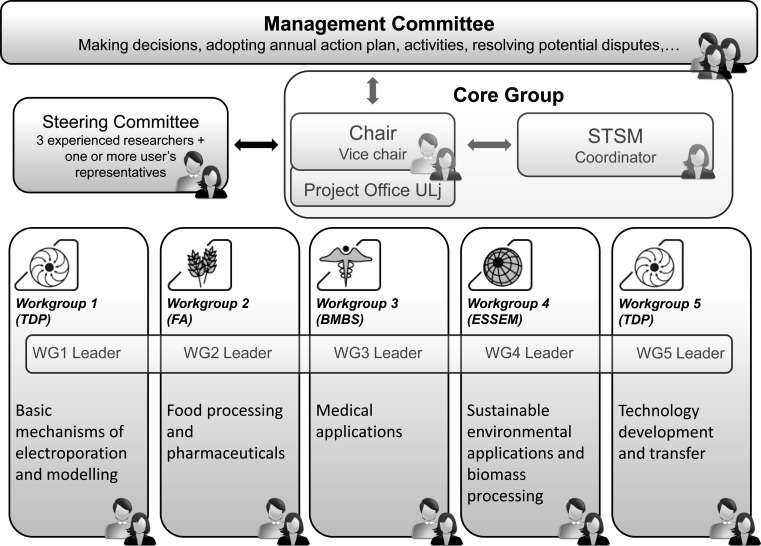
COST Action TD1104 management structure diagram identifying the key management entities and their interactions. *TDP* transdomain proposal, *FA* food and agriculture, *BMBS* biomedicine and molecular biosciences, *ESSEM* earth system science and environmental management, *STSM* short-term scientific mission

The research work in the COST action will be conducted by partners and financed predominantly through various research agencies and grants, while COST will provide the necessary coordination. The action is coordinated by the management committee (MC, Fig. 4). The MC supervises and coordinates implementation of the action. According to the rules of procedure for the MC, the chair and vice chair of the action were appointed at the kick-off meeting by the MC. The MC also established working groups (WGs) according to the scientific plan and appointed their leaders. The MC will meet once a year to set priorities, plan meetings and distribute funds to WG programs. The MC will summarize the WGs results and produce action reports, which will be posted on a dedicated Web site (www.electroporation.net).

Steering of the action will be further ensured by the steering committee, to which the MC appointed three experienced researchers from different WGs and at least one representative of potential users, e.g., an industry representative. A core group, composed of the chair, vice chair, WG leaders and short-term scientific missions (STSM) coordinator, will further ensure effective and continuous steering for the action by maintaining regular periodic contacts by means of conference calls, videoconferences and meetings every 4 months or more frequently when deemed necessary. The STSM coordinator will put emphasis on the effective use of STSM as an instrument of collaboration and training, with priority given to early-stage researchers, who will be encouraged to also apply for COST conference grants.

Five WGs have been established following the scientific plan: WG 1, Basic Mechanisms of Electroporation and Modeling; WG 2, Food Processing and Pharmaceuticals; WG 3, Medical Applications; WG 4, Sustainable Environmental Applications and Biomass Processing; and WG 5, Technology Development and Transfer. Specific objectives of each of these WG will be as follows

- *WG 1 Basic Mechanisms of Electroporation and Modeling* to obtain in-depth knowledge of the biological, physical and chemical mechanisms underlying electroporation
- *WG 2 Food Processing and Pharmaceuticals* to gain knowledge on process–product interactions in food and pharmaceutical applications involving electroporation and combine it with the latest findings in other application areas to improve equipment and process design
- *WG 3 Medical Applications* to disseminate present clinical applications, develop new applications and standardize clinical protocols
- *WG 4 Sustainable Environmental Applications and Biomass Processing* to improve the economic and technological efficiency of environmental and biomass applications of electroporation
- *WG 5 Technology Development and Transfer* to exchange technical knowledge on development of safe and reliable electroporation systems for industrial applications and clinical use.

Each WG will have a leader appointed by the MC who will be responsible for organization, program preparation and reporting to the MC. The organization of work and coordination in these five WGs will allow members to focus on specific scientific questions and research, facilitating coordination and preparation of annual programs and activities of the action, which will be prepared by the WGs and submitted for approval to the MC.

## Expected Results

Electroporation requires multidisciplinary research, integrating efforts of experts from different domains, from basic research to applications and from life sciences to engineering sciences. As experience from the past shows, interdisciplinary research, i.e., collaboration of a mixed group of experts from different fields, allows us to address even the most important world challenges, such as health issues, clean water, science and technology and energy sources.

Electroporation has a direct impact on health (cancer treatment, DNA vaccination) and environmental (water disinfection) issues. Huge benefits may be expected from applications in food preservation that increases nutritional value but also in reducing the waste from agricultural resources by improving extraction efficiency and reducing energy demand. Positive effects on the environment can also be expected in biomass processing (e.g., economic algae processing for use as an energy source) and wastewater treatment.

It is exactly through the mechanisms of networking and coordination activities provided within the COST TD1104 Action that synergies are expected, offering the possibility to improve the existing and develop new electroporation-based applications. These synergies will result in new spin-off project proposals, new processes and products achieved by means of international collaboration, multidisciplinary approach and reaching the critical mass in human resources and equipment. For example, in food processing optimization toward highest energy efficiency and low operation and investment costs is required to allow for further development of technology. Making use of the extensive amount of research performed in the medical sector, this COST action will allow a knowledge transfer and a significant step toward a broader use of electroporation in other industry sectors including the food processing industry and pharmacy.
